# Downhill running does not alter blood C1q availability or complement-dependent cytotoxicity of therapeutic monoclonal antibodies against haematological cancer cell lines *in vitro*

**DOI:** 10.1038/s41598-024-79690-8

**Published:** 2024-11-15

**Authors:** Harrison D. Collier-Bain, Frankie F. Brown, Adam J. Causer, Lois Ross, Daniela Rothschild-Rodriguez, Noah Browne, Rachel Eddy, Kirstie L. Cleary, Juliet C. Gray, Mark S. Cragg, Sally Moore, James Murray, James E. Turner, John P. Campbell

**Affiliations:** 1 Department for Health, University of Bath, Bath, BA2 7AY UK; 2 School of Applied Sciences, Edinburgh Napier University, Edinburgh, UK; 3 School of Biological Sciences, University of Southampton, Southampton, UK; 4 Antibody and Vaccine Group, Centre for Cancer Immunology, University of Southampton, Southampton, UK; 5 Department of Haematology, Royal United Hospitals Bath NHS Foundation Trust, Bath, UK; 6 School of Sport, Exercise and Rehabilitation Sciences, University of Birmingham, Birmingham, UK; 7 School of Medical and Health Sciences, Edith Cowan University, Perth, Australia

**Keywords:** Complement-dependent cytotoxicity, Immunotherapy, Exercise, Muscle damage, Complement C1q, Biochemistry, Biological techniques, Cancer, Immunology, Physiology, Biomarkers, Diseases, Oncology

## Abstract

Complement-dependent cytotoxicity (CDC) is a primary mechanism-of-action of monoclonal antibody (mAb) immunotherapies used to treat haematological cancers, including rituximab and daratumumab. However, mAb efficacy may be limited by reduced bioavailability of complement C1q – which activates the complement classical pathway following interactions with mAb-opsonised target cells. C1q is secreted by phagocytes upon recruitment to sites of muscle damage to facilitate muscular repair, hence we hypothesised that muscle damaging exercise may increase C1q ‘spill-over’ into blood. Additionally, other complement proteins (e.g., C1s) have been reported to increase following ultra-endurance and resistance exercise. Taken together, we hypothesised that muscle damaging exercise could be harnessed to enhance mAb-mediated CDC. In this study, *n* = 8 healthy males (28 ± 5-years) completed two 45-minute treadmill running protocols: (1) a flat running protocol at a speed 15% above anaerobic threshold, and (2) a downhill running protocol (− 10% slope) at the same speed. Blood samples were collected before, immediately after, and 1-hour, 24-hours, 2-days, and 4-days after exercise. Isolated serum was assessed for C1q by ELISA, and used to measure mAb (rituximab, daratumumab) mediated CDC against two haematological cancer cell lines (Raji, RPMI-8226) in vitro. Isolated plasma was assessed for markers of inflammation (C-reactive protein [CRP]), and muscle damage (creatine kinase [CK]) by turbidimetry. C1q and CDC activity were not different between running protocols and did not change over time (*p* > 0.05). Significantly greater perceived muscle soreness (*p* < 0.001) and fluctuations observed from baseline to 24-hours post-exercise in the downhill running trial in CK (+ 171%) and CRP (+ 66%) suggests some degree of muscle damage was present. It is possible that any increase in C1q post-exercise may have been masked by the increase and subsequent interaction with CRP, which utilises C1q to facilitate muscular repair. This is the first study to investigate whether exercise can increase circulating C1q and improve mAb-mediated CDC and our findings show that downhill running exercise does not increase circulating C1q nor improve CDC in vitro.

## Introduction

The development of monoclonal antibody (mAb) immunotherapy over the past two decades has revolutionised the treatment of haematological cancers and is considered a standard of care^1^. Several mAbs are now used to treat haematological cancers including the first mAb approved for oncological treatment, rituximab, which is an anti-CD20 mAb used to treat chronic lymphocytic leukaemia (CLL)^2^ and other B-cell haematological cancers. More recently, mAbs have become available for the treatment of other B-cell cancers, including daratumumab – an anti-CD38 mAb used to treat myeloma^3^.

The primary mechanisms-of-action for rituximab and daratumumab include antibody-dependent cellular cytotoxicity (ADCC), antibody-dependent cellular phagocytosis and complement dependent cytotoxicity (CDC)^4–6^. During an ADCC response, mAbs opsonise a specific antigen on the target cell surface allowing Fc𝛾 receptors (e.g. Fc𝛾IIIA/CD16a) on NK-cells and monocytes to interact with the Fc-portion of mAb-coated target cells. This results in cell death via exocytosis of perforins and granzymes^7,8^. We have shown that cycling at 15% above anaerobic threshold for ~ 30-minutes enhanced rituximab-mediated ADCC against autologous CLL cells ex vivo (129%)^9^. Additionally, we observed a trend towards greater ADCC post cycling exercise in the presence of autologous time-point matched plasma compared to heat-inactivated foetal calf serum (HI-FCS), which might be explained by the presence of complement proteins in autologous plasma. On the other hand, during a CDC response, complement component C1q interacts with the Fc-portion of mAb-opsonised target cells activating the classical complement pathway. Once activated, a proteolytic cascade of events occurs concluding with the assembly of a membrane attack complex (MAC) which alters cell permeability resulting in cell death^10^. Patients commonly exhibit a complete response following mAb-therapy, but disease eradication is uncommon. For example, rituximab and daratumumab achieve high complete remission rates, but persistence of minimal residual disease (MRD), and subsequent relapse, is inevitable^11–14^.

Interestingly, rituximab induces superior CDC activity compared to cellular-mediated effector mechanisms in vitro^15,16^ and in mouse models^17^. Daratumumab induces CDC activity similar to cellular-mediated mechanisms against primary myeloma cells in vitro^18^, and against myeloma cell lines in vitro^19^. As such, the efficacy of both of these mAbs can, at least in part, be attributed to CDC. One reason for CDC failure is reduced bioavailability of C1q^20,21^. To overcome this problem, patients with complement deficiencies have received rituximab therapy concomitantly with fresh-frozen plasma – containing complement proteins – resulting in enhanced rituximab activity^22,23^. The importance of C1q bioavailability to the efficacy of daratumumab is more pronounced in the setting of all-trans retinoic acid (ATRA), which upregulates surface CD38 expression and downregulates the expression of complement regulatory proteins such as CD55 and CD59, resulting in greater daratumumab-mediated CDC activity (+ 28% and + 20% cell lysis, respectively)^19,24^. Therefore, understanding and modifying complement kinetics in haematological malignancies provides an opportunity to improve the efficacy of mAb therapies that rely on CDC as a mechanism of action.

Complement system proteins are altered following exercise in healthy humans^25^, with proteins of the C1-complex (e.g. C1s) – important for classical pathway activation – increasing in the blood for up to three days following ultra-endurance and resistance exercise^26–28^. We have recently hypothesised that the elevation of complement proteins may be the result of increased monocyte/macrophage recruitment into damaged muscle^29^. In support of this, a study examining eccentric cycling for 30-minutes at 37% of concentric cycling maximal oxygen uptake (V̇O_2MAX_) in 13 healthy males was shown to increase the frequency of monocytes in blood, peaking 6-hours (+ 44.9%) and remaining elevated 24-hours (+ 16.2%) post-exercise. In addition, in muscle, expression of the macrophage marker CD163 was also shown to be increased, peaking at 48-hours^30^. Once monocytes have infiltrated muscle, their differentiation into M2-like macrophages facilitates the resolution of muscle injury^30–32^ and it has been shown that both monocytes and macrophages secrete C1q^33–35^. Therefore, it can be postulated that exercise-induced muscle damage may increase the intramuscular infiltration of monocytes and macrophages which secrete C1q that may ‘spill-over’ into blood 2- to 4-days post-exercise^36,37^ – alongside increases to other complement proteins in the cascade such as C1s^26–28^ – and thus, may improve the efficacy of mAb-mediated CDC. However, no study to date has investigated C1q kinetics in response to muscle damaging exercise and the effects of exercise on CDC responses in humans, and therefore further research is warranted.

In the present study we investigated the effects of downhill running – an established experimental method in humans for inducing muscle damage, previously shown to increase C-reactive protein (CRP)^38^ and creatine kinase (CK)^39–41^ – on serum C1q concentration and mAb-mediated CDC in vitro. We hypothesised that muscle damage would increase circulating C1q and, alongside other exercise-induced changes to the complement system, would enhance CDC of rituximab and daratumumab against haematological cancer cell lines in vitro^25,29^.

## Methods

### Participants

Eight healthy males (mean ± SD: age, 28 ± 5-years; height, 179.0 ± 7.2 cm; body mass, 73.4 ± 7.0 kg; body mass index, 22.9 ± 1.3 kg/m^2^; anaerobic threshold, 30.4 ± 5.4 mL⋅kg^− 1^⋅min^− 1^) provided written informed consent to participate in this study. All participants were between the ages of 18- to 35-years. Participants who regularly smoked, received medications, or reported health problems indicative of cardiovascular or inflammatory disease were excluded during screening. All experiments were performed in accordance with the Declaration of Helsinki and this study was approved by the Research Ethics Approval Committee for Health (REACH) at the University of Bath (EP 1920 036).

### Experimental design

**Fig. 1:**
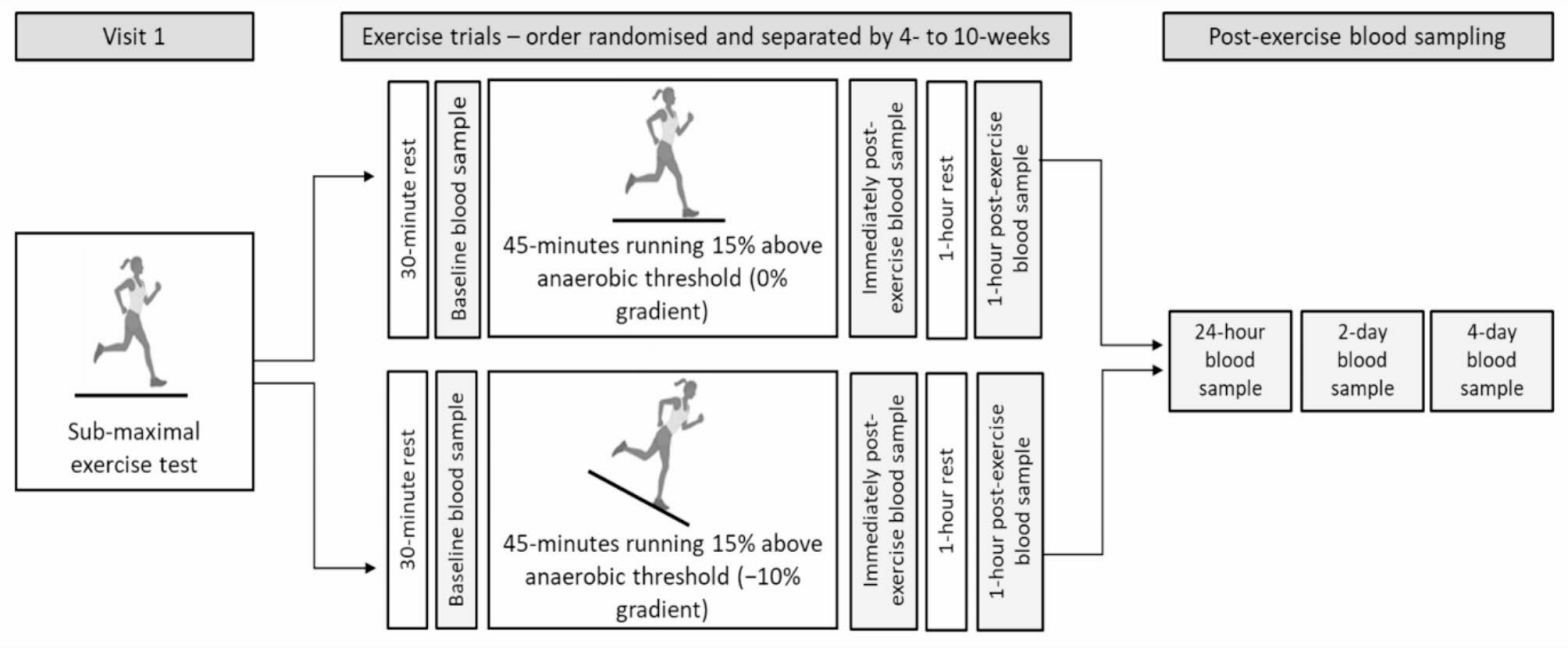
Schematic of the experimental procedures herein. Participants undertook a sub-maximal exercise test to a rating of perceived exertion of 17. Participants then completed a 45-minute bout of flat running (0% gradient) and downhill running (− 10% gradient) at a speed corresponding to 15% above their anaerobic threshold. Blood samples were collected following 30-minutes of rest and before exercise (baseline), immediately following exercise (within 3-minutes) and 1-hour following exercise. Participants were then invited back to laboratory for further post-exercise blood samples 24-hours following exercise, 2-days following exercise and 4-days following exercise.

Participants were asked to visit the laboratory on multiple occasions (Fig. 1). The first visit consisted of an anaerobic threshold test and was followed by two steady-state exercise trials (i.e., flat and downhill running) performed in a randomised order. The exercise trials consisted of continuous running for 45-minutes at a speed corresponding to 15% above individual anaerobic threshold on a treadmill (Saturn, HP Cosmos, Nussdorf-Traunstein, Germany) with the gradient set at 0% (flat) or − 10% (downhill). Participants returned to the laboratory 24-hours, 2-days, and 4-days following both exercise trials for post-exercise blood sampling. Participants arrived to the laboratory following an overnight fast and replicated dietary habits in the 24-hours before both trials. Exercise trials were performed at the same time of day to minimise diurnal variation in complement proteins^42^ and were separated by a minimum of 4-weeks and a maximum of 10-weeks.

### Exercise procedures

An incremental sub-maximal treadmill test determined anaerobic threshold in participants who were fasted for at least 4-hours, and abstained from alcohol, caffeine, anti-inflammatory medication, and exercise for 24-hours. The treadmill test started at 5.0 km⋅h^− 1^ and increased by 0.5 km⋅h^− 1^ every 30-seconds at a gradient of 0%, until participants achieved a rating of perceived exertion (RPE) of 17^43^. Gas exchange and ventilation (K5 Metalyser, COSMED, Italy), and heart rate (Garmin, USA) were recorded continuously during exercise, whilst RPE was recorded every 30-seconds. Pulmonary oxygen uptake (V̇O_2_), carbon dioxide production (V̇CO_2_), and ventilatory equivalents of O_2_ (V̇_E_/V̇O_2_) and CO_2_ (V̇_E_/V̇CO_2_) data were interpolated to 15-second averages. The V-slope method^44^ was used to determine anaerobic threshold – independently by two researchers –, and was further confirmed through visual inspection of V̇_E_/V̇O_2_ and V̇_E_/V̇CO_2_.

Participants underwent a further two bouts of exercise – separated by 4- to 10-weeks – between 7-days and 6-weeks following the anaerobic threshold test. For both visits, participants arrived at the laboratory following an overnight (≥ 10-hours) fast, whilst also having refrained from alcohol, caffeine, anti-inflammatory medications for 24-hours and exercise for 48-hours. Following 30-minutes of supine rest and a 5-minute warm-up at a self-selected speed on a treadmill, participants completed a 45-minute continuous bout of running at a speed that corresponded to 15% above their pre-determined anaerobic threshold, and at a gradient of either 0% (flat running) or − 10% (downhill running). Upon completion of the acute bouts of running, participants rested for a further 60-minutes. Delayed onset muscle soreness (DOMS) scores were recorded on a 10 cm visual analogue scale (VAS) 24-hours, 2-days, and 4-days post-exercise.

### Blood sampling and processing

During experimental trials, blood samples were collected at baseline and then at 0-hours, 1-hour, 24-hours, 2-days, and 4-days post-exercise. Using a 21-gauge needle (Becton & Dickinson, Oxford, UK), 25 mL of blood was drawn from an antecubital vein, with 5 mL collected into an ethylenediaminetetraacetic acid (EDTA) tube (K2E 6.0 mL, Becton & Dickinson, Oxford, UK), of which approximately 150 µL was used for the leukocyte differential and other haematological variables (Sysmex KX-21 N, Kobe, Japan). To minimise complement activation ex vivo, the remaining 20 mL of blood were distributed into two, sterile, plain glass vials (Kimble, Vineland NJ, USA)^25^.

EDTA treated blood was centrifuged (Heraeus Biofuge Primo R, Thermo Fisher Scientific, Loughborough, UK) within 15-minutes of collection at 2,000 × g, 4 °C for 15-minutes. Blood collected for the isolation of serum were stirred with an aseptic wooden spatula, left to clot at room temperature for 30- to 60-minutes, and subsequently centrifuged at 900 × g, 4 °C for 20-minutes. Plasma and serum were immediately aspirated and aliquoted into cold, sterile glass vials, and stored at − 80 °C for later analysis.

### Quantification of plasma and serum proteins

Creatine kinase (CK) and C-reactive protein (CRP) were quantified in plasma using turbidimetry (CK-NAC and hsCRP, respectively; Randox, RX Daytona, Crumlin, UK). Serum C1q concentrations were determined using enzyme-linked immunosorbent assay (ELISA) (Hycult Biotech Incorporated, Plymouth Meeting, PA, USA) following manufacturer guidelines. Data quantified from plasma and serum isolated from blood samples collected after the baseline time-point were adjusted for plasma volume changes using haematocrit and haemoglobin via an automated haematology analyser (Sysmex KX-21 N, Kobe, Japan)^45^.

### Cell Culture

The effects of exercise on complement-dependent cytotoxicity (CDC) were assessed in two haematological cancer cell line settings including, Raji (CD20^+^, lymphoma; ECACC 85011429), RPMI-8226 (CD38^+^, myeloma; ECACC 87012702) – purchased from the UK Health Security Agency – and RPMI-8226 cells which were also treated with all-trans retinoic acid (Sigma-Aldrich, Missouri, USA) (ATRA-8226; CD38^++^) for 48-hours prior to assays. Raji and RPMI-8226 cells were cultured in medium containing glutamine enriched RPMI-1640 (Gibco™, MA, USA), supplemented with 10% (v/v) HI-FCS (Gibco™, MA, USA), 1% (v/v) penicillin/streptomycin (Thermo Fischer Scientific, Loughborough, UK), and 1% (v/v) sodium pyruvate (Gibco™, MA, USA). ATRA-8226 were cultured in the same medium supplemented with 2 µM/mL of ATRA, which as described earlier is used to upregulate CD38, and downregulate CD55 and CD59 on the target cell surface^19,24^. All cell lines were maintained at 5 × 10^5^ cells/mL, 5% CO_2_, 37 °C and passaged into fresh medium every 2-days.

### Complement-dependent cytotoxicity (CDC) assay

To analyse the effects of exercise on CDC against haematological cancer cell lines, 2 × 10^6^ cells of each cell line were labelled with the membrane permeable molecule, calcein acetoxymethyl ester (calcein-AM) (Invitrogen™, Thermo Fisher Scientific, Loughborough, UK) following manufacturers protocol. Briefly, this method relies on the principle that calcein-AM passively diffuses across the target cell membrane into the cell cytoplasm where hydrolysis by intracellular esterases converts it to calcein, a fluorescent dye. Calcein is unable to diffuse back across the membrane and is retained in the cytoplasm until cell lysis occurs. Following the removal of excess calcein-AM through washing, the amount of calcein released is proportional to the amount of cell lysis.

Serum samples from each time-point per participant were thawed at 4 °C the morning of each assay and diluted 1:1 with cold phosphate buffered saline (PBS; KCl 0.2 g/L, KH_2_PO_4_ 0.2 g/L, NaCl 8.0 g/L, Na_2_HPO_4_ 1.15 g/L; without CaCl_2_ and MgCl_2_ herein) (Dulbecco’s PBS, Sigma Aldrich, Kent, UK) in sterile, cold, glass vials and kept on ice. Then, 40 µL of diluted serum were added to respective wells of a non-treated, U-shaped-bottom, 96-well plate (Falcon™, Thermo Fischer Scientific, Loughborough, UK) and placed on ice. Serum from a resting sample was heat-inactivated for 30-minutes at 57 °C, diluted 1:1 with cold PBS and used in lieu of normal human serum in spontaneous lysis (negative control) and maximum lysis (positive control) conditions. Anti-CD20 mAb rituximab (final concentration: 0.1 µg/mL; Selleckchem, TX, USA), anti-CD38 mAb daratumumab (final concentration: 10 µg/mL; Janssen, UK), or PBS – used for antibody-independent CDC controls – at a volume of 50 µL were added to respective wells (excluding spontaneous and maximum lysis conditions) before labelled Raji, RPMI-8226, and ATRA-8226 cells were seeded at 5 × 10^3^ cells per well in 25 µL PBS. PBS was added to ensure all wells achieved a final volume of 200 µL. Maximum lysis replicates remained as a 100 µL volume during incubation after which time 100 µL of lysis buffer was added to achieve a 200 µL final volume, as discussed next. Plates were incubated for 45-minutes in the dark at 37 °C, 5% CO_2_.

Following incubation, 100 µL of lysis buffer (4% Triton X-100; Invitrogen™, Thermo Fisher Scientific, Loughborough, UK) was added to maximum lysis replicates and plates were centrifuged at 100 × g for 2-minutes. Next, 75 µL of acellular supernatant from each well was transferred to a non-treated flat-bottom, black, 96 well plate (Corning™, Thermo Fischer Scientific, Loughborough, UK) and fluorescence (485 nm, 530 nm) was measured using a Pherostar plate reader (BMG Labtech, Ortenberg, Germany) with the gain – based on positive controls –, and the focal height optimised per plate. Relative fluorescence units produced were converted into a percentage of specific lysis using the following equation:

$$\% {\rm{ }}Specific\ {\rm{ }}Lysis{\rm{ }} = {\rm{ }}\left( \begin{array}{l}\left( {Sample{\rm{ }}-{\rm{ }}Spontaneous{\rm{ }}\left[ {target{\rm{ }}cells{\rm{ }}alone} \right]} \right){\rm{ }} \div {\rm{ }}\\\left( {Triton{\rm{ }}X - 100{\rm{ }}-{\rm{ }}Spontaneous{\rm{ }}\left[ {target{\rm{ }}cells{\rm{ }}alone} \right]} \right)\end{array} \right){\rm{ }} \times {\rm{ }}100$$

Experimental conditions were seeded in triplicate, and spontaneous and maximum conditions were seeded in six replicates. All procedures involved in CDC assays were undertaken in aseptic conditions.

### Statistical analysis

Statistical analysis was carried out using SPSS (IBM SPSS Statistics Version 28, IL, USA). Data are presented as means ± SD unless otherwise stated. Repeated measures analysis of variance (ANOVA) with Bonferroni corrected pairwise comparisons – where significant effects were observed – were performed to determine main effects of condition (flat running, downhill running), time (baseline, immediately post-, 1-hour post-, 24-hours post-, 2-days post-, 4-days post-exercise), and condition*time interactions for all parameters except for DOMS scores which were analysed using the same repeated measures ANOVA but for time-points 24-hours, 2-days, and 4-days post-exercise only, and data for assessing exercise intensity that was averaged across the exercise portion of the trial. Paired sample t-tests (following confirmation of parametric distribution via a Shapiro-Wilk test) were used to determine differences between conditions for data assessing exercise intensity averaged across the exercise trial. Effect sizes from repeated measures ANOVA were reported as partial eta squared (ηp^2^). Effect sizes were determined small (ηp^2^ = 0.01), medium (ηp^2^ = 0.06), or large (ηp^2^ = 0.14)^46^. Statistical significance was accepted at *p* ≤ 0.05. Occasional missing data are reflected in the reported degrees of freedom.

## Results

### Characteristics of exercise

All participants completed one bout of flat running and one bout of downhill running. Anaerobic threshold occurred at a V̇O_2_ of 30.4 ± 5.4 mL⋅kg^−1^⋅min^−1^, corresponding to a speed of 8.1 ± 1.2 km⋅hr^−1^. Table 1 presents results from the exercise trials, including speed corresponding to + 15% of anaerobic threshold (km⋅hr^−1^), V̇O_2_ (mL⋅kg^−1^⋅min^−1^), relative V̇O_2_ as a percentage of anaerobic threshold (%), V̇CO_2_ (mL⋅kg^− 1^⋅min^−1^), respiratory exchange ratio, heart rate (bpm), heart rate as a percentage of age-predicted maximum (%), and RPE (Borg 6–20 scale). During flat running trials, participants ran approximately 13% above anaerobic threshold, whilst during downhill running trials, participants ran below anaerobic threshold (− 16%), which is expected due to the lower aerobic cost running downhill.

**Table 1:** Characteristics of exercise during flat running and downhill running conditions. Data are mean ± SD, *n* = 8.

	Flat running	Downhill Running
Speed (km⋅hr^− 1^)	9.4 ± 1.3	9.4 ± 1.3
V̇O_2_ (mL⋅kg^−1^⋅min^−1^)	33.6 ± 5.8	25.0 ± 4.9^***^
V̇O_2_ (% anaerobic threshold)	112.9 ± 25.7	84.3 ± 19.7^***^
V̇CO_2_ (mL⋅kg^−1^⋅min^−1^)	27.9 ± 4.8	20.7 ± 4.2^**^
Respiratory exchange ratio	0.83 ± 0.06	0.82 ± 0.07
Heart rate (bpm)	152 ± 23	134 ± 23^*^
Heart rate (% age predicted max)	78.8 ± 12.4	69.9 ± 12.1^*^
Rating of perceived exertion	14 ± 1	13 ± 1

^*^indicates a significant difference at *p* < 0.05, ^**^indicates a significant difference at *p* < 0.01, ^***^indicates a significant difference at *p* < 0.001.

### Delayed onset muscle soreness (DOMS) following treadmill running

**Fig. 2:**
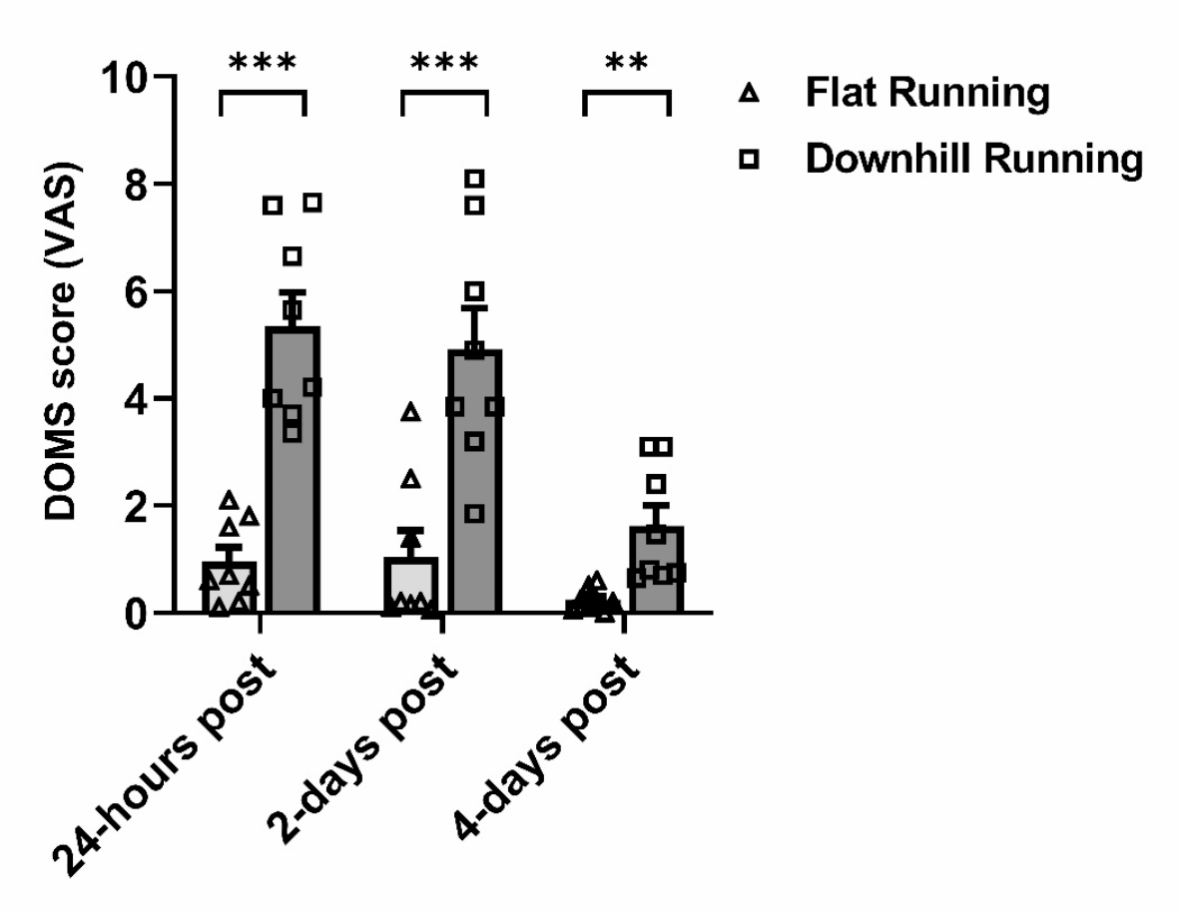
Delayed onset muscle soreness (DOMS) reported on a 10 cm visual analogue scale (VAS) following flat running (light grey bars, triangle data points) and downhill running (dark grey bars, square data points) trials. ^**^indicates a significant difference between running conditions at *p* < 0.01, ^***^indicates a significant difference between running conditions at *p* < 0.001, following *post hoc* Bonferroni comparisons. Data are mean ± SEM, *n* = 8.

A significant main effect of condition (*F*_(1,7)_ = 67.50, *p* < 0.001, ηp^2^ = 0.91), time (*F*_(2,14)_ = 38.94, *p* < 0.001, ηp^2^ = 0.85), and condition*time interaction (*F*_(2,14)_ = 21.33, *p* < 0.001, ηp^2^ = 0.75) were observed for DOMS scores. Self-reported DOMS scores were greater following downhill running when compared to flat running at 24-hours (+ 463%, *p* < 0.001), 2-days (+ 371%, *p* < 0.001), and 4-days (+ 583%, *p* = 0.003) post-exercise (Fig. 2).

### Plasma volume changes in response to treadmill running

Plasma volume was not different between conditions (*F*_(1,6)_ = 0.001, *p* = 0.98, ηp^2^ < 0.001) but significantly changed over time (*F*_(5,30)_ = 4.90, *p* = 0.002, ηp^2^ = 0.45), with no interaction effects (*F*_(1.79,10.75)_ = 1.10, *p* = 0.38, ηp^2^ = 0.16) (Table 2). Plasma volume increased significantly from 0-hours to 1-hour (+ 14%, *p* = 0.002), 24-hour (+ 10%, *p* = 0.002), and 2-days (+ 16%, *p* = 0.037) following downhill running with no changes observed following flat running. These changes were used to correct data quantified from plasma and serum samples.

**Table 2:** Haemodynamic variables from baseline to 4-days post-exercise in flat running and downhill running conditions, with results from repeated measures ANOVA. _a_Values reflect the summation of monocytes, basophils, and eosinophils. Data are mean ± SD, *n* = 8.

	Running condition	Baseline	0-hour	1-hour	24-hour	2-day	4-day	Effect of condition	Effect of time	Interaction effect
**Plasma volume (% whole blood)**	Flat	58.1 ± 2.1	56.8 ± 1.6	58.4 ± 1.5	57.8 ± 2.6	58.0 ± 2.9	59.7 ± 2.4	*F*_(1,6)_ = 0.001, *p* = 0.98, ηp^2^ < 0.001	*F*_(5,30)_ = 4.9, *p* = 0.002, ηp^2^ = 0.45	*F*_(1.79,10.75)_ = 1.1, *p* = 0.36, ηp^2^ = 0.16
	Downhill	57.6 ± 4.1	55.7 ± 2.4	58.4 ± 2.2^††^	57.7 ± 2.6^††^	58.8 ± 2.3^†^	58.5 ± 2.1			
**Blood Lactate (mmol/L)**	Flat	0.8 ± 0.2	1.9 ± 0.9	1.0 ± 0.4	0.8 ± 0.3	0.9 ± 0.5	0.7 ± 0.2	*F*_(1,7)_ = 5.08, *p* = 0.059, ηp^2^ = 0.42	*F*_(2.00,13.98)_ = 8.97, *p* = 0.003, ηp^2^ = 0.56	*F*_(1.69,11.82)_ = 2.93, *p* = 0.099, ηp^2^ = 0.30
	Downhill	0.9 ± 0.4	1.1 ± 0.5	0.7 ± 0.3	1.0 ± 0.3	0.8 ± 0.3	0.7 ± 0.3			
**Blood Glucose (mmol/L)**	Flat	5.4 ± 0.2	5.7 ± 0.4	4.9 ± 0.4	5.1 ± 0.4	5.1 ± 0.5	4.7 ± 1.0	*F*_(1,7)_ = 0.14, *p* = 0.72, ηp^2^ = 0.02	*F*_(5,35)_ = 4.08, *p* = 0.005, ηp^2^ = 0.37	*F*_(5,35)_ = 1.64, *p* = 0.18, ηp^2^ = 0.19
	Downhill	5.1 ± 0.3	5.3 ± 0.5	4.9 ± 0.4	4.9 ± 0.4	5.4 ± 0.6	5.0 ± 0.5			
**Leukocytes (×10** ^**9**^ **/L)**	Flat	5.02 ± 1.29	6.85 ± 2.08	5.57 ± 2.12	5.05 ± 1.46	4.49 ± 0.92	4.56 ± 0.96	*F*_(1,6)_ = 1.4, *p* = 0.28, ηp^2^ = 0.19	*F*_(5,30)_ = 11.51, *p* < 0.001, ηp^2^ = 0.66	*F*_(5,30)_ = 1.23, *p* = 0.32, ηp^2^ = 0.17
	Downhill	5.01 ± 1.33	6.38 ± 1.82^*^	5.25 ± 1.52^†^	4.74 ± 1.23	4.70 ± 1.13^†^	5.05 ± 1.36			
**Lymphocytes (×10** ^**9**^ **/L)**	Flat	1.52 ± 0.33	2.34 ± 0.74	1.27 ± 0.20	1.48 ± 0.29	1.44 ± 0.36	1.42 ± 0.20	*F*_(1,6)_ = 0.01, *p* = 0.92, ηp^2^ = 0.002	*F*_(1.26,7.55)_ = 13.23, *p* = 0.006, ηp^2^ = 0.69	*F*_(2.01,12.04)_ = 1.43, *p* = 0.28, ηp^2^ = 0.19
	Downhill	1.58 ± 0.21	2.13 ± 0.49	1.34 ± 0.26^†^	1.47 ± 0.21	1.47 ± 0.17^†^	1.48 ± 0.27			
**Mixed**_**a**_ **(×10** ^**9**^ **/L)**	Flat	0.51 ± 0.14	0.59 ± 0.21	0.44 ± 0.13^†^	0.44 ± 0.12	0.45 ± 0.10	0.46 ± 0.13	*F*_(1,6)_ = 0.36, *p* = 0.57, ηp^2^ = 0.06	*F*_(5,30)_ = 9.09, *p* < 0.001, ηp^2^ = 0.60	*F*_(2.00,11.97)_ = 1.44, *p* = 0.28, ηp^2^ = 0.19
	Downhill	0.52 ± 0.18	0.56 ± 0.16	0.51 ± 0.17	0.43 ± 0.14	0.44 ± 0.13	0.50 ± 0.16			
**Neutrophils (×10** ^**9**^ **/L)**	Flat	3.01 ± 1.09	3.93 ± 1.48	3.85 ± 1.99	3.13 ± 1.20	2.60 ± 0.74	2.68 ± 0.79	*F*_(1,6)_ = 0.47, *p* = 0.52, ηp^2^ = 0.07	*F*_(5,30)_ = 6.77, *p* < 0.001, ηp^2^ = 0.53	*F*_(1.84,10.89)_ = 0.38, *p* = 0.67, ηp^2^ = 0.06
	Downhill	2.90 ± 1.02	3.70 ± 1.29^*^	3.59 ± 1.53	2.84 ± 0.99	2.80 ± 0.95	3.08 ± 1.30			
**Platelets (×10** ^**9**^ **/L)**	Flat	215 ± 28	292 ± 55^*^	215 ± 26^†^	216 ± 31^†^	213 ± 31^†^	219 ± 35^†^	*F*_(1,6)_ = 4.06, *p* = 0.09, ηp^2^ = 0.40	*F*_(1.70,10.20)_ = 28.65, *p* < 0.001, ηp^2^ = 0.83	*F*_(5,30)_ = 2.50, *p* = 0.053, ηp^2^ = 0.29
	Downhill	212 ± 32	277 ± 45^**^	222 ± 37^††^	227 ± 37^†^	223 ± 29	230 ± 36			

^*^indicates significant difference from Baseline at *p* < 0.05, ^**^indicates a significant difference from baseline at *p* < 0.01, ^†^indicates a significant difference from 0-hours post-exercise at *p* < 0.05, ^††^indicates a significant difference from 0-hours post exercise at *p* < 0.01 following *post hoc* Bonferroni comparisons. Occasional missing data is reflected in the reported degrees of freedom. Due to whole blood count data missing from a baseline sample for one participant, all data corrected for plasma volume change are *n* = 7. ANOVA, analysis of variance.

### C-reactive protein (CRP) changes in response to treadmill running

**Fig. 3:**
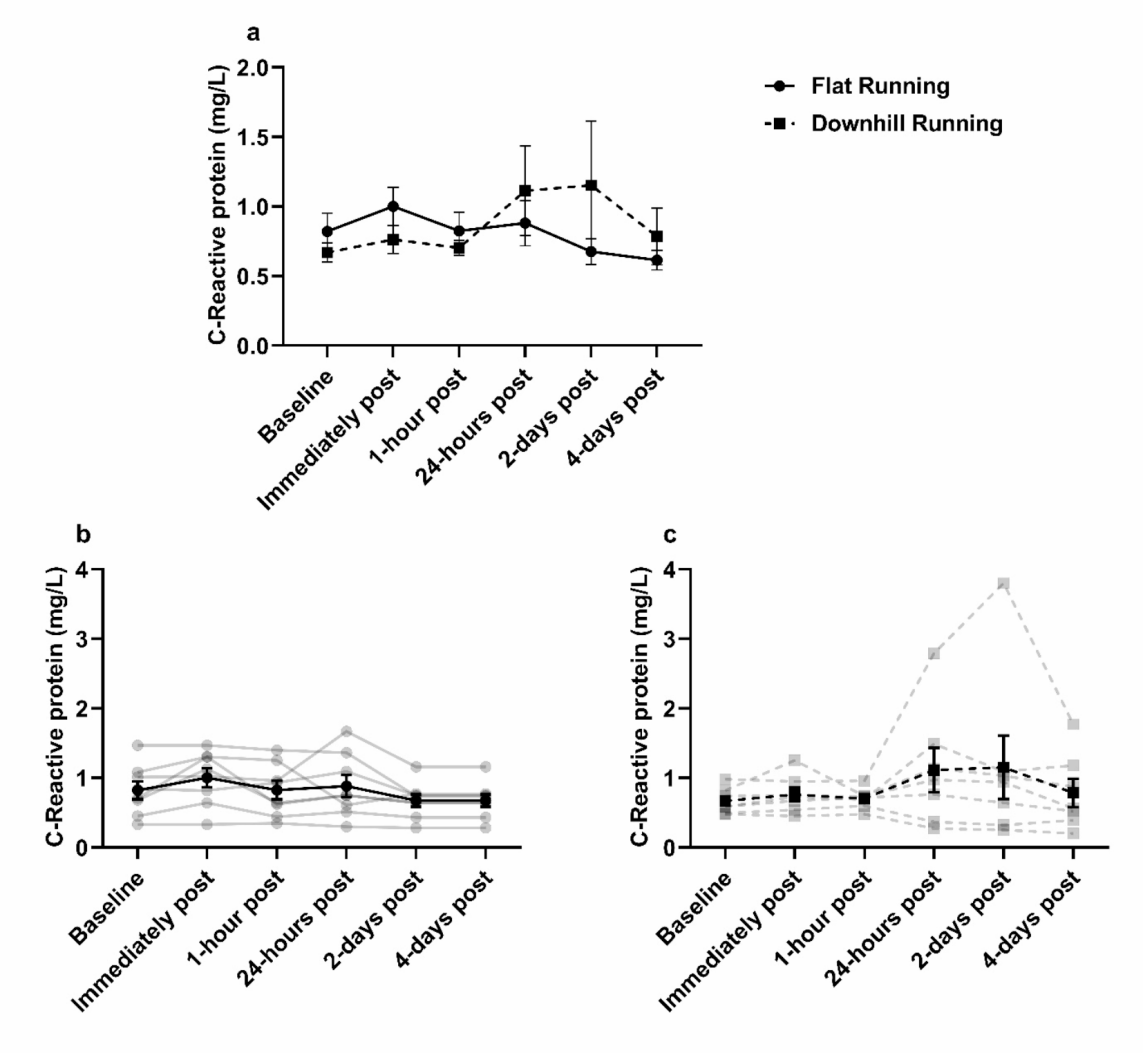
Plasma C-reactive protein concentration. **(a)** C-reactive protein was unchanged between flat and downhill running with no significant changes over time, *p* > 0.05. **(b)** Individual responses (transparent lines) and mean (solid black line) during flat running. **(c)** Individual responses (transparent lines) and mean (solid black line) during downhill running. Data are mean ± SEM, *n* = 8.

No significant main effects of condition (*F*_(1,6)_ = 0.14, *p* = 0.72, ηp^2^ = 0.02), or time (*F*_(1.29,7.75)_ = 0.98, *p* = 0.38, ηp^2^ = 0.14), but a significant interaction effect was observed for plasma CRP (*F*_(5,30)_ = 3.41, *p* = 0.015, ηp^2^ = 0.36) (Fig. 3). There were fluctuations observed for CRP 24-hours and 2-days (+ 66% and + 72%, respectively) following downhill running before returning to baseline.

### Creatine kinase (CK) changes in response to treadmill running

**Fig. 4:**
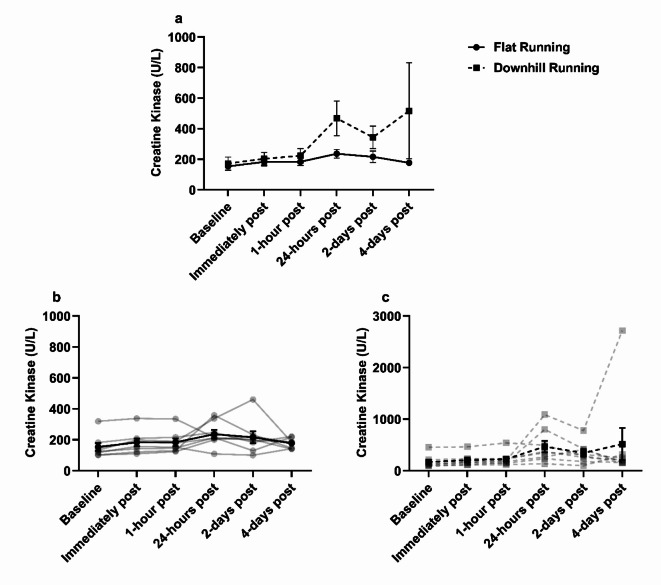
Plasma Creatine Kinase concentration. **(a)** Creatine kinase was not different between flat and downhill running conditions and did not significantly change over time, *p* > 0.05. **(b)** Individual responses (transparent lines) and mean (solid line) during flat running. **(c)** Individual responses (transparent lines) and mean (solid line) during downhill running. Data are mean ± SEM, *n* = 8.

No significant main effects of condition (*F*_(1,7)_ = 2.38, *p* = 0.17, ηp^2^ = 0.25), time (*F*_(1.33,9.29)_ = 1.51, *p* = 0.26, ηp^2^ = 0.18), or interaction (*F*_(1.09,7.64)_ = 1.02, *p* = 0.35, ηp^2^ = 0.13) were observed for plasma CK concentration (Fig. 4). CK concentrations remained stable from baseline to 1-hour post exercise, after which, there was a fluctuation observed from baseline to 24-hours post flat running (+ 54%), and from baseline to 24-hours and 4-days post downhill running (+ 171% and + 200%, respectively).

### Complement C1q changes in response to treadmill running

**Fig. 5:**
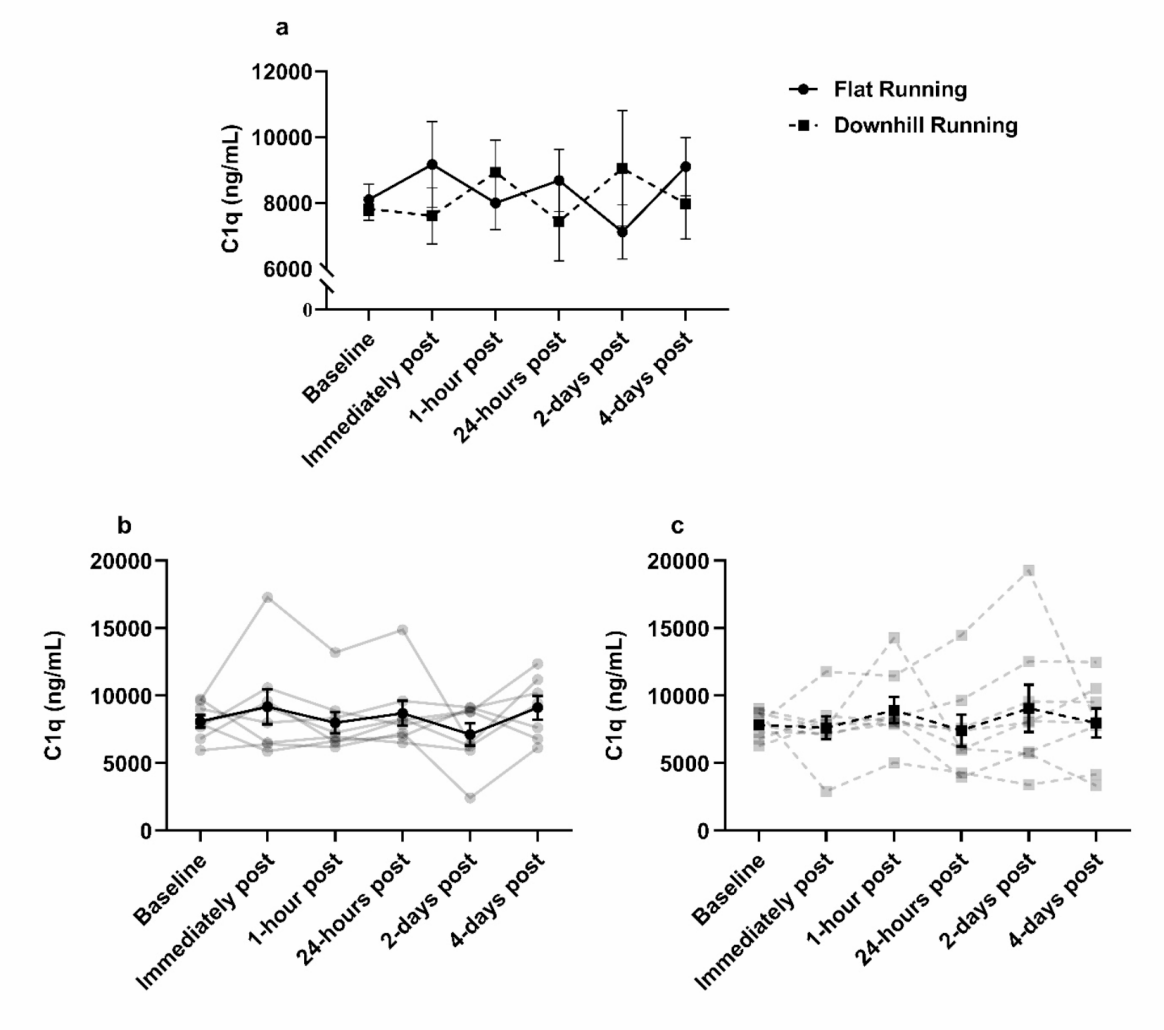
Serum C1q concentration during flat and downhill running trials. **(a)** C1q was not significantly different between flat and downhill running or between time-points, *p* > 0.05. **(b)** individual responses (transparent lines) and mean (solid line) during flat running. **(c)** individual responses (transparent lines) and mean (solid line) during downhill running. Data are mean ± SEM, *n* = 8.

The effects of flat and downhill running on blood C1q are displayed in Fig. 5. ANOVA revealed no significant main effects of time (*F*_(5,35)_ = 0.19, *p* = 0.96, ηp^2^ = 0.03), condition (*F*_(1,7)_ = 0.19, *p* = 0.97, ηp^2^ = 0.01), or interaction effect (*F*_(5,35)_ = 1.37, *p* = 0.26, ηp^2^ = 0.16) on C1q concentration. C1q concentration appeared to fluctuate across the blood sampling sessions, with the greatest fluctuation observed from baseline to 2-days (+ 15.7%) post downhill running. Individual variance in C1q concentrations is shown in Fig. 5b and c.

### Complement dependent cytotoxicity (CDC) changes in response to treadmill running

**Fig. 6:**
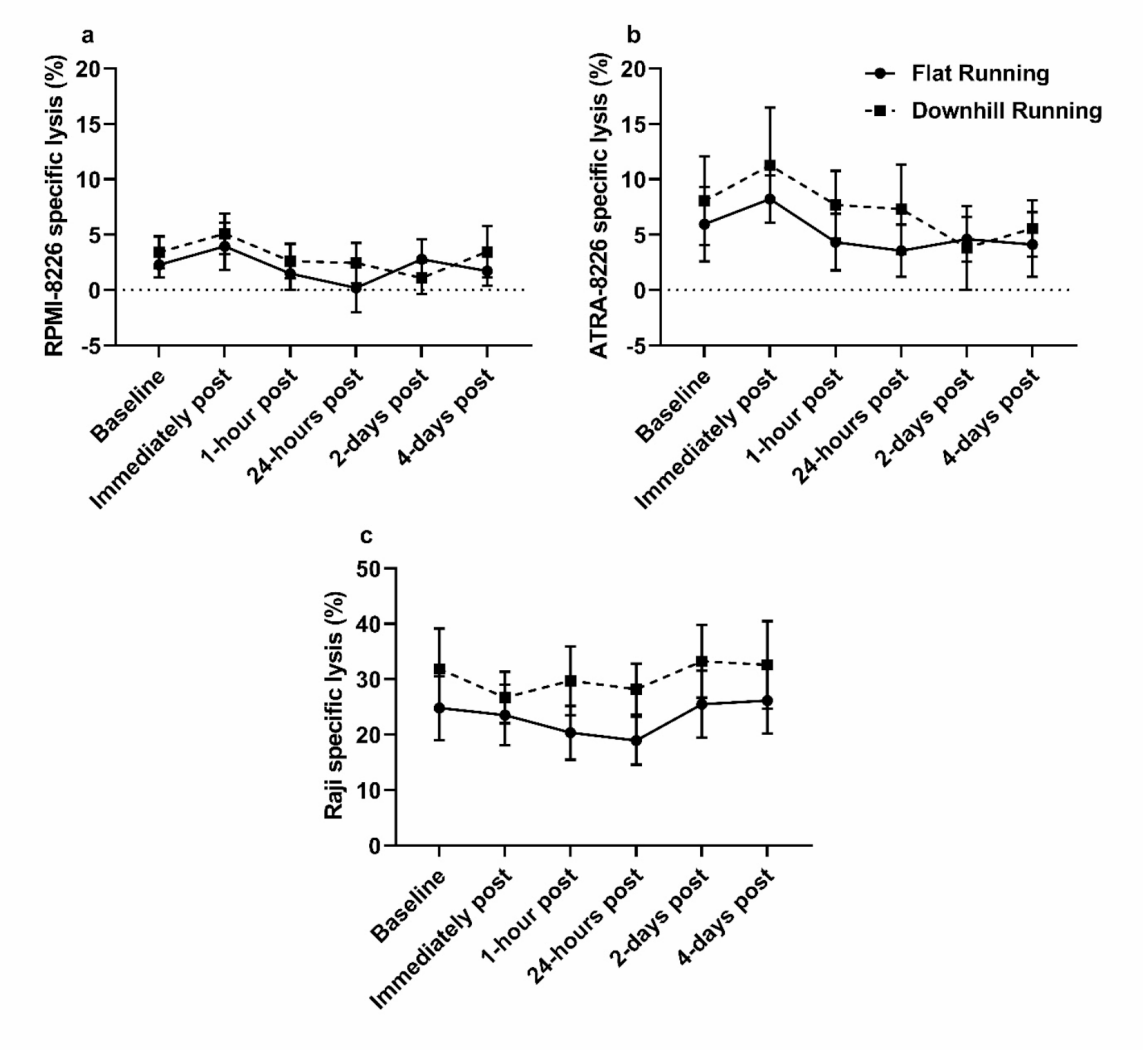
Monoclonal antibody-mediated CDC against haematological cancer cell lines. **(a)** Daratumumab-mediated CDC against RPMI-8226 cells was unchanged between flat running and downhill running, and across time points following *post hoc* Bonferroni comparisons, *p* > 0.05, *n* = 8. **(b)** Daratumumab-mediated CDC against ATRA-8226 cells was unchanged between flat running and downhill running, and across time points following *post hoc* Bonferroni comparisons (*p* > 0.05), but was consistently higher than untreated RPMI-8226, *p* > 0.05, *n* = 7. **(c)** Rituximab-mediated CDC against Raji cells was unchanged between flat and downhill running, and across timepoints, *p* > 0.05, *n* = 8. Data are mean ± SEM.

Daratumumab-mediated CDC against RPMI-8226 and ATRA-8226 is shown in Fig. 6A and B, respectively. ANOVA revealed a significant main effect of time for RPMI-8226 and ATRA-8226 (*F*_(2.95,20.68)_ = 4.05, *p* = 0.021, ηp^2^ = 0.37 and *F*_(1.67,10.02)_ = 8.07, *p* = 0.01, ηp^2^ = 0.57, respectively), with no effects of condition (*F*_(1,7)_ = 1.32, *p* = 0.29, ηp^2^ = 0.16 and *F*_(1,6)_ = 2.30, *p* = 0.18, ηp^2^ = 0.28, respectively) or interaction (*F*_(2.37,16.57)_ = 1.83, *p* = 0.19, ηp^2^ = 0.21 and *F*_(5,30)_ = 0.92, *p* = 0.48, ηp^2^ = 0.13 respectively). Lysis of RPMI-8226 and ATRA-8226 was similar across both exercise modalities, with fluctuations observed immediately post-exercise in flat running (+ 73% and + 38%, respectively) and downhill running (+ 49% and + 40%, respectively) trials. ATRA-8226 cell lysis appeared consistently higher than RPMI-8226 during flat running and downhill running trials, but this difference was not statistically significant (*F*_(1,6)_ = 0.64, *p* = 0.45, ηp^2^ = 0.10 and *F*_(1,6)_ = 1.05, *p* = 0.35, ηp^2^ = 0.15, respectively).

Rituximab-mediated CDC against Raji cells is shown in Fig. 6C. A significant main effect of time (*F*_(2.20,15.42)_ = 3.66, *p* = 0.047, ηp^2^ = 0.34) and condition (*F*_(1,7)_ = 7.72, *p* = 0.027, ηp^2^ = 0.53) was observed for Raji cell lysis. Raji cell lysis remained relatively unchanged overtime but was significantly greater in downhill running trials compared to flat running trials 1-hour and 24-hours post exercise (*p* = 0.041 and *p* = 0.02, respectively). However, no interaction effect was observed (*F*_(5,35)_ = 0.83, *p* = 0.53, ηp^2^ = 0.11) and thus, significant differences 1-hour and 24-hours post exercise may be driven by baseline differences between running conditions (*p* = 0.056).

### Leukocyte kinetics during treadmill running

Leukocyte kinetics are displayed in Table 2. A significant effect of time was observed for leukocytes (*F*_(5,30)_ = 11.51, *p* < 0.001, ηp^2^ = 0.66), lymphocytes (*F*_(1.26,7.55)_ = 13.23, *p* = 0.006, ηp^2^ = 0.69), mixed cells (reflects the summation of monocytes, basophils, and eosinophils) (*F*_(5,30)_ = 9.09, *p* < 0.001, ηp^2^ = 0.60), neutrophils (*F*_(5,30)_ = 6.77, *p* < 0.001, ηp^2^ = 0.53), and platelets (*F*_(1.70,10.20)_ = 28.65, *p* < 0.001, ηp^2^ = 0.83). Leukocytes (*p* = 0.048), and neutrophils (*p* = 0.038) were significantly elevated immediately post downhill running, whilst platelets were significantly elevated immediately post-exercise in both flat running and downhill running trials (*p* = 0.031 and *p* = 0.004, respectively) before returning to normal concentrations 1-hour post-exercise and thereafter.

## Discussion

The primary objective of this study was to determine if downhill running increased blood C1q and enhanced rituximab- and daratumumab-mediated CDC activity against two haematological cancer cell lines in vitro. A secondary aim was to assess muscle damage and inflammation induced by downhill running. Circulating C1q fluctuated around baseline levels in the hours and days after both flat and downhill running with no differences observed between trials. Additionally, exercise had no effect on CDC activity against haematological cell lines in vitro.

Few studies have investigated the effects exercise-induced muscle damage on blood C1q concentration. Previous research in mouse models shows that serum C1q is significantly elevated by ~ 50 µg/mL following cryo-induced muscle damage^47^. Although in contrast with our findings, this disparity may be due to the difference in the severity of muscle damage induced. Specifically, the muscle damage induced previously consisted of a liquid nitrogen dipped probe applied directly to exposed muscle of mice^47^. Although C1q kinetics have not been investigated in the presence of muscle damage induced by an individual bout of exercise in humans, two studies have investigated C1q following individual bouts of aerobic exercise^48,49^. It was reported that C1q was reduced immediately following 20-minutes of high intensity treadmill running in trained (− 5.2%), and untrained (− 6.4%) prepubescent females aged 10- to 12-years^48^. Another study reported no change in C1 proteins following 10- to 20-minutes of a moderate-intensity cycling test in healthy humans^49^. Our findings are consistent with the latter, however they are not comparable with the former study given the differences in age and biological sex^48^, particularly given that there is an age-associated increase to circulating C1q, linked to sarcopenia^50^. It is possible that any increase in intramuscular or circulating C1q following downhill running herein may have been used by CRP to facilitate in muscle repair^51,52^, as previously purported^25^. This is supported by a study showing that following 12-weeks of descending stair walking (eccentric exercise) plasma C1q is reduced by − 51%, in 30 obese, sedentary females^53^, indicating that a degree of muscle damage is required for C1q reduction, consistent with resistance training interventions^50^.

The fluctuations observed in CRP from baseline to 24-hours and 2-days post downhill running herein (+ 66% and + 72%, respectively) in addition to the fluctuations observed in CK from baseline to 24-hours following downhill running (+ 176%) – indicating some degree of muscle damage was present – provides further support to the argument above. However, these fluctuations were largely driven by one individual. Indeed, the elevated CK from baseline to 24-hours following downhill running herein was far less in our study when compared with previous findings (%Δ from baseline to 24-hours post downhill running = + 171% vs. + 710%, respectively)^41^, likely due to a more vigorous exercise intensity utilised previously (80% of V̇O_2_ maximum) and that on average, participants V̇O_2_ herein were below anaerobic threshold (− 16%) when running downhill which might equate to an intensity < 60% of V̇O_2_ maximum^54^.

Analogous to the C1q response, mAb-mediated CDC of haematological cancer cell lines was unchanged following either running protocol. CDC mediated by rituximab and daratumumab relies on the binding of C1q to the mAbs Fc-portion following opsonisation of target cells^9^. Thus, the observation of no change in C1q could support the lack of change observed in CDC activity, however, it is important to acknowledge that other proteins required for classical complement activation were not measured herein. It was observed that ATRA-8226 cell lysis was consistently higher (~ 5%) than RPMI-8226, which aligns with previous findings^19^. The authors suggested that the non-significant increase in CDC may be the result of a smaller relative increase in the expression of CD38 induced by ATRA treatment when compared to other myeloma cell lines such as, XG1^19^.

These results have clinical implications. We have recently suggested that individual bouts of muscle damaging exercise such as downhill running or resistance training, could be harnessed as a way of improving the efficacy of mAb-mediated CDC in vivo^25,29^. However, our findings show that muscle damaging exercise provides no additional benefit to mAb efficacy, and therefore, future research should focus on other ways in which exercise may be used as an adjuvant to mAb therapy^29^. For example, we have shown that cycling for 30-minutes at an intensity 15% above anaerobic threshold increases the frequency of natural killer (NK)-cells in the blood of patients with CLL, resulting in an enhancement of rituximab-mediated ADCC against autologous CLL cells ex vivo^9^.

It is important to acknowledge that C1q requires other complement proteins (e.g., C1s) to activate the classical complement pathway^55^. Indeed, previous research has demonstrated increased C1s concentrations for up to three days following ultra-endurance and resistance exercise – which may induce a degree of muscle damage –^26–28^. Thus, it can be speculated that an increase to C1s concentration occurred herein, however, this was not measured in our study. We sought to focus on C1q because of its clinical relevance to mAb immunotherapy and the limited research investigating C1q kinetics in response to muscle damaging exercise in humans. Nevertheless, future research should quantify various complement protein changes in response to downhill running. Another limitation to this study is that the intensity and/or duration of exercise may not have been sufficient to induce a substantial degree of muscle damage to cause significant changes in CK as seen previously in downhill running models^39–41^. Indeed, previous studies have increased the speed of the treadmill during downhill running to induce a similar physiological response as flat running to investigate the effects of muscle damaging exercise on blood lymphocytes^41^. In the present study, despite no change in CK, the differences found in self-reported DOMS scores (1.38–4.40 VAS, Fig. 2) between flat and downhill running indicate that the procedures induced significant stress on the lower limb musculature. The concentration of C1q pre-exercise and over time in flat and downhill running trials was heterogenous, but no trends specific to downhill running were observed for our primary outcome measures – C1q, and CDC activity.

In summary, this is the first study to investigate C1q kinetics, and examine the efficacy of rituximab- and daratumumab-mediated CDC against haematological cell lines following muscle damaging exercise in humans in vitro. Our findings show that blood C1q was unchanged in response to downhill or flat running, and there was no effect of exercise on mAb-mediated CDC in vitro.

## Data Availability

The datasets generated during and/or analysed during the current study are available from the corresponding author on reasonable request.
